# Fluorescence polarization immunoassay for the determination of diclofenac in wastewater

**DOI:** 10.1007/s00216-020-03058-w

**Published:** 2020-11-25

**Authors:** Anna Raysyan, Robin Moerer, Bianca Coesfeld, Sergei A. Eremin, Rudolf J. Schneider

**Affiliations:** 1 Bundesanstalt für Materialforschung und -prüfung (BAM), 12200 Berlin, Germany; 2 Department of Chemistry, Humboldt-Universität zu Berlin, 10099 Berlin, Germany; 3 Chemical Faculty, M.V. Lomonosov Moscow State University, Moscow, Russian Federation 119991; 4 Technische Universität Berlin, Straße des 17. Juni 135, 10623 Berlin, Germany

**Keywords:** Diclofenac, FPIA, Water, Wastewater, NSAID

## Abstract

Pharmacologically active compounds are often detected in wastewater and surface waters. The nonsteroidal anti-inflammatory drug diclofenac (DCF) was included in the European watch list of substances that requires its environmental monitoring in the member states. DCF may harmfully influence the ecosystem already at concentrations ≤ 1 μg L^−1^. The fast and easy quantification of DCF is becoming a subject of global importance. Fluorescence polarization immunoassay (FPIA) is a homogeneous mix-and-read method which does not require the immobilization of reagents. FPIA can be performed in one phase within 20–30 min, making it possible to analyse wastewater without any complicated pre-treatment. In this study, new tracer molecules with different structures, linking fluorophores to derivatives of the analyte, were synthesized, three homologous tracers based on DCF, two including a C_6_ spacer, and one heterologous tracer derived from 5-hydroxy-DCF. The tracer molecules were thoroughly assessed for performance. Regarding sensitivity of the FPIA, the lowest limit of detection reached was 2.0 μg L^−1^ with a working range up to 870 μg L^−1^. The method was validated for real wastewater samples against LC-MS/MS as reference method with good agreement of both methods.

**Supplementary Information:**

The online version contains supplementary material available at 10.1007/s00216-020-03058-w.

**Figure:**
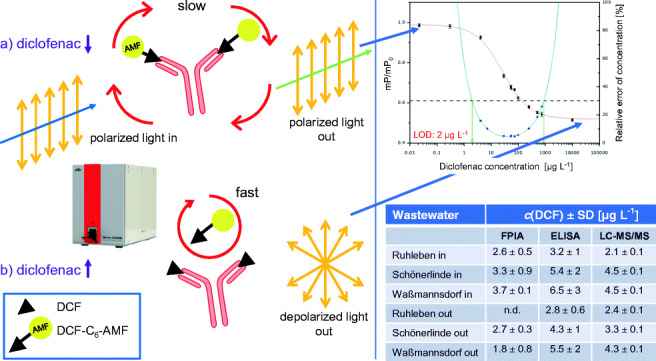
Graphical abstract

## Introduction

Diclofenac (DCF) is a nonsteroidal anti-inflammatory drug (NSAID) with analgesic, anti-inflammatory, and antipyretic properties. The mechanism of action of diclofenac, like that of other NSAIDs, involves inhibition of cyclooxygenase (COX-1 and COX-2). Another pharmacological effect is the inhibition of prostaglandin synthesis in vitro [1]. The disposal of DCF via treated wastewater may harmfully influence the ecosystem already at concentrations ≤ 1 μg L^−1^ [2]. Therefore, DCF was included in the first EU Water Framework Directive Watch List for emerging water pollutants to be monitored in surface waters of the EU member states [2, 3]. Various instrumental methods have been applied for detection of DCF, including ultra-performance liquid chromatography (UPLC) coupled to high-resolution mass spectrometers [4, 5]. However, these techniques are expensive and require extensive sample preparation [6].

Immunoassays have been studied and used for the detection of DCF, and many research reports deal with polyclonal and monoclonal antibody production [5, 7–9] and the development of heterogeneous immunoassay methods, such as the established ELISA [10, 11] and ULISA [12] and automatic flow-based plasmonic ELISA [13] methods, which both need immobilization of one binding partner and several washing steps. Due to these time-consuming steps, they are not suitable for the demand of a fast, efficient, and high-throughput screening method. A homogeneous immunoassay, such as the fluorescence polarization immunoassay (FPIA), does not require the immobilization of reagents; it can be performed in one phase and is not in need of any washing steps, which makes the entire procedure faster [14–16]. This was the incentive to develop a sensitive FPIA method for the determination of DCF.

Performing an FPIA is simple; it is just mixing the reagents, so FPIA is sometimes classified as a mix-and-read assay. The signal originates from observing the fluorescence emission of an analyte-fluorophore conjugate (the tracer) [17]. It is added to the sample and the mixture excited by plane-polarized light, produced via a polarizer between lamp and sample cuvette. The emitted fluorescence radiation is recorded via a photomultiplier in front of which another polarizer is positioned. An intensity value of the incident light is recorded (“parallel”, *I*_║_) and then a second value after rotating the second polarizer by 90° (“perpendicular”, *I*_**⊥**_). Some instruments possess a second detector in 90° position which records *I*_**⊥**_ simultaneously. The difference of both recordings divided by the sum of both recordings (in the case of equal sensitivity in both directions) is called fluorescence polarization FP (reported in millipolarization units, mP). Now, antibody is added in solution. Two extreme examples may illustrate the processes that take place. If (a) no (or a small amount of) analyte is present in the solution, the larger fraction of the tracer binds to the antibody. As a result, its orientation in space, which before was in steady change due to Brownian motion, rotation and diffusion, is rather conserved: the registered intensity values of both detectors gradually decline while differing strongly from each other and after reading at a fixed time, a large polarization FP is registered. If (b) there is a high analyte concentration in the mixture, mostly analyte molecules bind to the antibody added, the tracer remains largely “free” (and, therefore, subject to Brownian motion, fast rotation, and diffusion) so that in consequence the incident polarized light is transformed to depolarized fluorescence emission in all directions: little light hits the detector and *I*_**⊥**_ is similar to *I*_║_, resulting in a small difference, i.e. a small value for the polarization FP. Plotting FP against the analyte concentration on a logarithmic scale results in a sigmoidal curve as with all immunoassays [18, 19]. As fluorophore, in the large majority of applications, fluorescein is used, and the instruments are adapted to its peak excitation at 494 nm and peak emission at 521 nm for measurement.

According to the assay steps, FPIA is a technique where kinetics play an important role. Therefore, the length of all incubation steps (i.e. the time after addition of the tracer and before addition of the antibody, and the time after which the read-out is recorded) has to be optimized in order to obtain a sensitive assay and controlled in order to obtain good reproducibility. Moreover, since FPIA is a homogeneous assay, no washing steps are performed, in contrast to ELISA, and sample constituents are in contact with the antibody and tracer for prolonged time. Lastly, sample constituents that might interfere with the fluorescence measurement, be it via own fluorescence of by quenching, have to be evaluated for their effect on assay performance.

In this study, new tracer molecules, linking the fluorescein fluorophore with or without a spacer to the diclofenac moiety, were synthesized and assessed for performance in the FPIA. A C_6_ spacer derivative of diclofenac, diclofenac aminohexanoic acid amide (DCF-Ahx), had previously been synthesized by Schmidt et al. [20].

## Materials and methods

### Chemicals and materials

A monoclonal antibody against diclofenac (clone 12G5) (mouse IgG), described in Huebner et al. [10], was kindly provided by Dietmar Knopp (Technische Universität München): 200 μL of 5 mg mL^−1^ mAb anti-DCF 12G5, in buffered solution (50 mM Na_2_HPO_4_, 20 mM NaH_2_PO_4_, Tris-HCl 0.1 M, pH 7.4, azide 0.02%). *N*-Hydroxysuccinimide (NHS), *N,N′-*dicyclohexylcarbodiimide (DCC), chlorotrimethylsilane (TMS-Cl), abs(olute) *N,N-*dimethylformamide (DMF), diclofenac sodium salt (DCF), 5-hydroxy diclofenac (5-OH-DCF), 4′-hydroxy diclofenac (4′-OH-DCF), aceclofenac, chloroform, triethylamine, and sodium azide were purchased from Merck KGaA (Darmstadt, Germany). 4′-(Aminomethyl)fluorescein hydrochloride (AMF) was from Invitrogen (Carlsbad, CA, USA), methanol (MeOH) from J.T. Baker (Griesheim, Germany), and ethanol from ChemSolute (Renningen, Germany). Ultrapure reagent water for buffers and solutions was obtained from a Milli-Q Synthesis A10 water purification system (Merck Millipore, Darmstadt, Germany). All FPIA experiments were carried out in borate buffer, 50 mM, pH 8.5, with 0.1% sodium azide. TLC sheets (2.5 × 7.5 cm; silica gel 60 with concentration zone/without fluorescence indicator) were from Merck.

## Methods

### Reference analysis by LC-MS/MS

DCF reference concentrations of samples were determined by LC-MS/MS using an Agilent 1260 Infinity LC system with a binary pump, degasser, autosampler, and column heater. The chromatographic separation was carried out on a Kinetex XB-C18, 100 Å, 2.6 μm, 150 × 3 mm analytical LC column with an UHPLC C18, 3 mm guard column (both Phenomenex, Aschaffenburg, Germany). As mobile phases, Milli-Q water with 10 mM NH_4_Ac and 0.1% (*v*/*v*) acetic acid (A) and MeOH with 10 mM NH_4_Ac and 0.1% (*v*/*v*) AcOH (B) were used. The system was run at a flow rate of 350 μL min^−1^ and a column heater temperature of 30 °C. An elution gradient was applied, starting with 80% A for the first 15 min. Within 5 min, A is decreased to 5% (95% B). Then, A is ramped up back to 80% within 0.5 min and maintained at this level for 14.5 min to re-equilibrate the column. Fifteen microliters of sample was injected. Mass spectrometric detection was performed on an ABSciex 6500 Triple Quad mass spectrometer. Electrospray ionization (ESI) in positive ionization mode was employed.

### Tracer synthesis

**Fig. 1:**
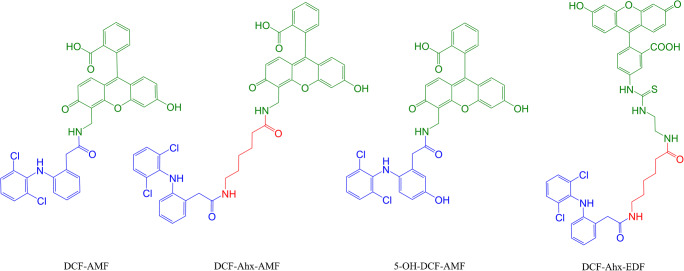
Chemical structures of the synthesized tracers

Three tracers based on diclofenac (homologous tracers) with and without spacer and an additional tracer based on 5-OH-DCF (heterologous tracer) were synthesized (Fig. 1). The fluorescent tags were coupled to the haptens by the NHS activated ester method developed by Eremin and co-workers [21, 22] with minor modifications (details in the Electronic Supplementary Material (ESM) esp. Figs. S1––S3).

### Fluorescence polarization immunoassay

#### Equipment

Fluorescence polarization was determined on a Sentry 2000Si (Ellie LLC, Germantown, WI, USA), a multi-well fluorescence polarization instrument equipped with a ceramic fluid metering system pump for automated reagent dosage. The light source is an LED with 485/535-nm filter set and a 510-nm dichroic mirror. Reactions are read in black medium-binding 8-microwell Fluorotrac strips (Greiner, Frickenhausen, Germany) (12-microwell strips can also be read).

#### Preparation of calibrators

A stock solution (ca. 10 g L^−1^) of DCF was prepared gravimetrically by dissolving an appropriate amount in ethanol. Working standards were prepared by diluting the stock solution either in borate buffer or in Milli-Q water and were stored in amber glass vials at 4 °C.

#### Running the assay

One hundred twenty microliters of borate buffer, 40 μL of Milli-Q water (blank) or sample, and 30 μL of an appropriate dilution of the tracer stock (tracer working solution, TWS) are pipetted into each microwell of an 8-well strip and the blank value (FP_0_) read after a certain tracer incubation time (see below). Thirty microliters of an appropriate antibody dilution is added to this mix. After shaking for the optimal antibody incubation time (see below), FP is determined.

#### Optimization of sensitivity

Optimization of the FPIA for each individual tracer means to find the optimum ratio between antibody and tracer (i.e. dilution and volume) to obtain maximum sensitivity at a reasonable signal/noise ratio, to result in a low variance. This was achieved in 3 steps: (1) First, the amount of tracer was initially set as low as possible with a still acceptable signal fluctuation. Intensity and FP are observed over time to select an appropriate time. (2) Next, antibody titration was performed by running the assay with 40 μL Milli-Q water to mimic a sample. Thirty microliters of different antibody dilutions was added to this solution to analyse for tracer binding. (3) Finally, FPIA calibration curves were recorded. Again, 120 μL borate buffer, 40 μL of calibrators, and 30 μL of the TWS were mixed before 30 μL of the selected antibody dilution was added.

FPIA is a kinetic assay in which the degree of polarization changes over time; equilibrium is not completely reached during the desired short incubation times. Therefore, the time for the tracer to equilibrate with the system (tracer incubation time) and the time allowed for competition of the tracer and the analyte to bind to the antibody (antibody incubation time) had to be individually evaluated and mixing and shaking are very important for the assay reproducibility.

Initial to all measurements, the FP of the blank (FP_0_) was determined and later on all FP values read divided by FP_0_. For calibration, the results were plotted against the logarithm of the DCF concentrations, and to the data points, a sigmoidal curve described by a logistic, four-parameter equation, was fitted, using Origin 8G Software (OriginLab, Northampton, MA, USA). To evaluate the sensitivity of the different FPIAs, precision profiles according to Ekins [23] were constructed, using, for interpolation of the profile, the model by Hoffmann et al. [24]. Limits of detection (LOD) were determined allowing for a relative error of the determined concentration of 30%.

#### Cross-reactivity

The specificity of the monoclonal antibody in the FPIA, employing DCF-Ahx-AMF as tracer, was evaluated by determining the cross-reactivity (CR_%_) [25] of structurally similar compounds (5-OH-DCF, 4′-OH-DCF, aceclofenac), by determining their IC_50_ values and calculating their CR_%_ as follows:

$$ {\mathrm{C}\mathrm{R}}_{\%}=\frac{\mathrm{I}{\mathrm{C}}_{50}\ \left(\mathrm{DCF}\right)}{{\mathrm{I}\mathrm{C}}_{50}\ \left(\mathrm{test}\ \mathrm{compound}\right)}\times 100\% $$

## Results and discussion

### Optimization of FPIA and comparison of tracers

In this work, new tracers were synthesized. The strategy for tracer synthesis, namely, to use the amino group in AMF and EDF and coupling DCF via its carboxylic acid group, especially compared to using, in two cases, 6-aminohexanoic acid (6-Ahx) as spacer, was to study the influence of tracer structure on the assay sensitivity. The structures of the tracers are shown in Fig. 1.

The combination of tracer and antibody has a significant influence on sensitivity, selectivity, and reliability of an FPIA, and should always be carefully studied. The intensity of the blank (borate buffer) on the Sentry 2000Si instrument is about 37,000 in both orientations (*I*_║_ and *I*_**⊥**_); FP is close to zero (0 mP). When a tracer dilution of 1:10,000 is added, intensity reading rises to ca. 200,000. This ca. 5-fold increase is a prerequisite for stable readings with the instrument and can be achieved by increasing the tracer concentrations. In any case, signal development has to be observed and the optimal tracer incubation time, before the addition of the antibody, determined. This resulted in waiting times of 2.5–6 min (see Table 1). Moreover, it was found that tracer dilutions must be freshly prepared every day.

**Table 1:** Characteristics of the binding of the four tracers to the monoclonal anti-DCF antibody 12G5 (diluted accordingly in borate buffer; tracer dilution: 1:10,000)

Tracer	Optimal antibody dilution	Concentration of antibody in optimal dilution (μg mL^−1^)	FP value of free tracer (mP)	Optimal tracer incubation time (s)	Optimal antibody incubation time (s)
DCF-AMF	1:400	12.5	221	300	90
DCF-Ahx-AMF	1:800	6.25	114	240	90
DCF-Ahx-EDF	1:800	6.25	133	270	80
5-OH-DCF-AMF	1:350	14.3	72	150	10

**Fig. 2:**
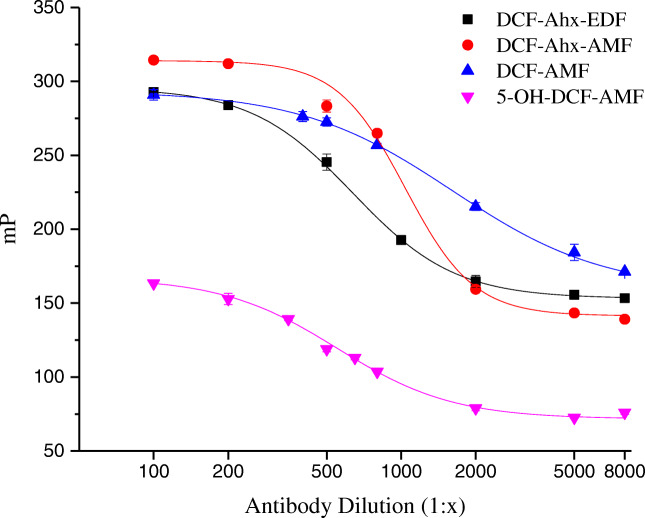
Antibody titration curves with the 4 tracers

When the antibody is added and FP determined, a tracer that effectively binds to the antibody leads to a strong decrease in FP with increasing dilution of the antibody (antibody titration) in a sigmoidal course (Fig. 2). When the tracer cannot bind to the antibody, no change with antibody concentration is observed.

It can be derived from Fig. 2 that all four tracers synthesized bind to the antibody. The optimum dilution of the antibody can be obtained from the antibody dilution curves in Fig. 2; it corresponds to approximately 50% of the maximum signal. Higher dilutions result in too small signal changes, lower dilutions waste antibody. Optimum dilutions were in the range of 1:350 to 1:800 (Table 1).

In FPIA, antibody binding is kinetically controlled; thus, the time until measurement after adding the antibody must be optimized individually. For the homologous tracers, an antibody incubation time of 90 s mostly suited all assays. The only exception was for the heterologous tracer 5-OH-DCF-AMF, where results obliged to choose a significantly shorter incubation time of 10 s (Table 1).

### Sensitivity and measurement range

**Fig. 3:**
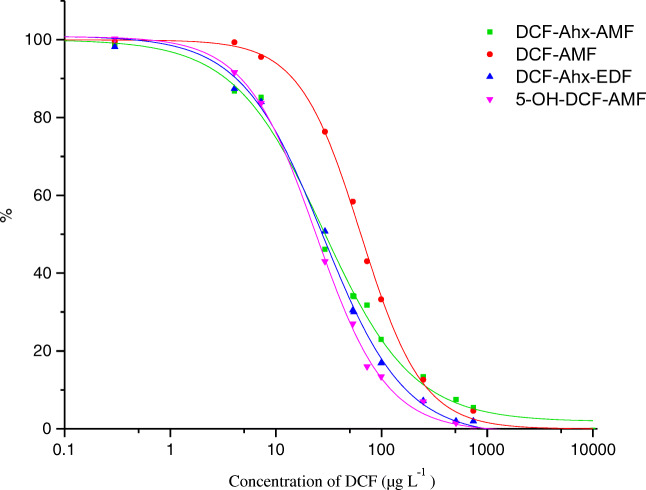
Calibration curves obtained for optimized conditions with the four tracers

A calibration curve for each individual tracer was recorded using the optimized conditions in Table 1 and diclofenac calibrators between 2·10^−2^ and 10^4^ μg L^−1^ were employed. The curves are shown in Fig. 3.

It is a clear advantage of the Sentry 2000Si instrument that it allows for serial measurement of 8 or 12 wells in contrast to single cuvette instruments. Moreover, the sensitivity of its photomultiplier is excellent. So, an optimization trial with 8 concentrations can be done in one single run.

**Fig. 4:**
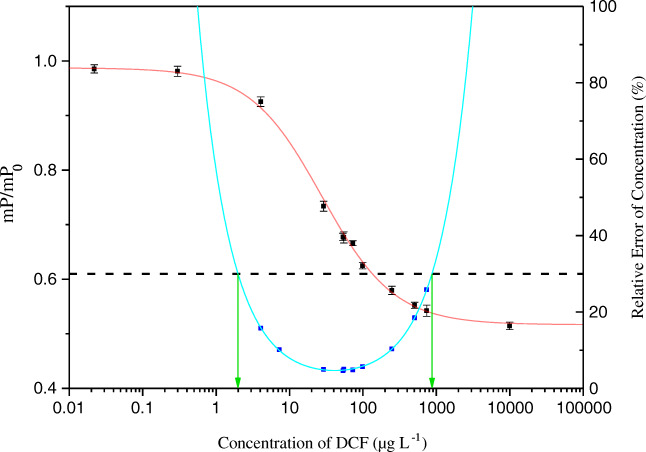
FPIA calibration curve with tracer DCF-Ahx-AMF (red solid line), precision profile (blue squares and cyan line), and measurement range (indicated by green arrows) from 2.0 to 870 μg L^−1^, determined via intersection points at 30% allowed relative error of the determined concentration (black dotted line)

The measurement range was assessed applying the concept of the precision profile. A function, $y=\left(a\times {x}^b\right)+c+\left(\frac{d}{x^e}\right)$, developed before [24], allows to fit a continuous line to the data points of the precision profile [26], with *a*, *b*, *c*, *d*, and *e* being variables and *x* the concentration of the analyte. With this function, it is possible to accurately calculate the measurement range shown exemplarily for DCF-Ahx-AMF in Fig. 4. A relative error of the determined concentration of 30% was considered allowable to mark the lower limit of detection (LOD) and the upper limit of detection. The individual measurement ranges for the four tracers are given in Table 2.

**Table 2:** Measurement ranges and respective IC_50_ achieved with the 4 different tracers

Tracer	Measurement range (μg L^−1^)	IC_50_ (μg L^−1^)
DCF-AMF	8.3–550	63
DCF-Ahx-AMF	2.0–870	28
DCF-Ahx-EDF	3.7–500	28
5-OH-DCF-AMF	2.2–260	24

The best tracer for obtaining a sensitive FPIA for DCF was DCF-Ahx-AMF, producing a very low LOD of 2.0 μg L^−1^ and exhibiting a good calibration correlation with a coefficient of determination (*r*^2^) of 0.9976 (*n* = 3). The corresponding IC_50_ was 28 μg L^−1^; the working range extended to 870 μg L^−1^.

With the heterologous tracer 5-OH-DCF-AMF, approximately the same sensitivity as with the homologous tracer DCF-Ahx-AMF was obtained (compare Table 2). According to some studies [27–29], heterologous tracers may improve the sensitivity. Yet, many tracers built on heterologous structures will not bind to the antibody (see [30]). A preliminary guess if a tracer will bind—before performing its synthesis—is to determine the cross-reactivity of its underlying hapten in ELISA. A structure with cross-reactivity of 10% and higher can often be used to prepare a functional heterologous tracer [27]. Naturally, this is an approximate value and it is necessary to assess the tracer in the envisaged assay. In our case, 5-OH-DCF had a cross-reactivity of 13% in an ELISA using the same antibody [10]. It could be proven that the tracer built upon it was functional, but it did not allow for a significant improvement of sensitivity.

On the *y*-axis, for comparability reasons, a normalized polarization is plotted which results from setting the value of the upper asymptote of each curve to 100% and the value of each lower asymptote to 0% and recalculating FP accordingly.

### Cross-reactivity of the antibody

Diclofenac is metabolized in the body to two major metabolites, predominantly 4′-hydroxy diclofenac (4′-OH-DCF), and 5-hydroxy diclofenac (5-OH-DCF), respectively, two compounds that could end up in wastewater, too. One other structurally related pharmaceutical compound, aceclofenac, was also tested for binding since it is also used as a pharmaceutical [10]. As tracer in this study, only DCF-Ahx-AMF was used. The obtained cross-reactivity values are given in Table 3.

**Table 3:**
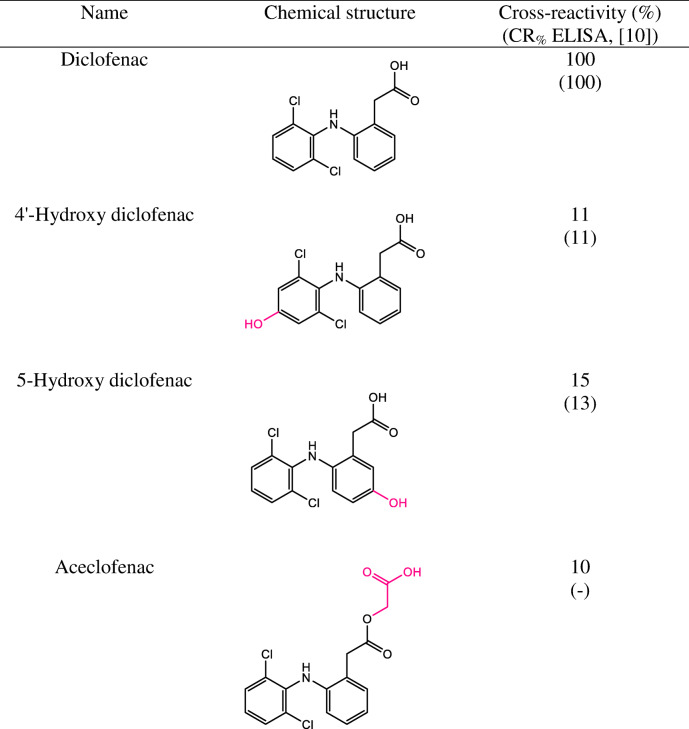
Cross-reactivity (CR) of the antibody, determined by FPIA. Antibody: anti-DCF mAb 12G5; Tracer: DCF-Ahx-AMF

On average, the monoclonal antibody 12G5 characterized by Huebner et al. via ELISA [10] showed a CR lower than 10%, except for 4′-OH-DCF and 5-OH-DCF. In this work, we checked these two compounds and their CR proved to be, within the limits of error, the same. Cross-reactivity of aceclofenac was not studied then. We here determined a cross-reactivity of 10%.

### Analysis of real wastewater samples

Six wastewater samples were obtained from three different wastewater treatment plants from Berlin (Ruhleben) and Brandenburg (Schönerlinde, Waßmannsdorf). The samples were first filtered through folded paper filters and then through glass-fibre syringe filters. Total organic carbon content (TOC) and pH were determined (cf. ESM Table S2). Influent samples had 3–5 times higher TOC values than effluent samples, pH was around 7.4, except sample 6, an effluent sample, which had a pH of 6.8. Influent samples were diluted 1:100 with Milli-Q water before analysis, and effluent samples 1:10. The comparison of results for the wastewater samples, analysed by three methods, is shown in Table 4.

**Table 4:** Concentration of diclofenac (DCF) in six wastewater samples determined by FPIA, ELISA [31], and the described LC-MS/MS method

No.	Wastewater treatment plant, influent/effluent	*c* (DCF) ± SD (μg L^−1^)		
		FPIA	ELISA [31]	LC-MS/MS
1	Ruhleben, influent	2.6 ± 0.5	3.2 ± 1	2.1 ± 0.1
2	Schönerlinde, influent	3.3 ± 0.9	5.4 ± 2	4.5 ± 0.1
3	Waßmannsdorf, influent	3.7 ± 0.1	6.5 ± 3	4.5 ± 0.1
4	Ruhleben, effluent	n.d.	2.8 ± 0.6	2.4 ± 0.1
5	Schönerlinde, effluent	2.7 ± 0.3	4.3 ± 1	3.3 ± 0.1
6	Waßmannsdorf, effluent	1.8 ± 0.8	5.5 ± 2	4.3 ± 0.1

The coefficient of variation of FPIA measurement replicates (*n* = 3) ranged from 3 to 44% (average: 21%; highest value was associated with sample no. 6 with a concentration determined close to the LOD), being mostly lower than for ELISA (average: 32%), so precision was considered satisfactory for a fast and direct measurement of wastewater samples in the low microgram per liter range. The FPIA and LC-MS/MS results are in good agreement with each other. One effluent sample could not be determined by FPIA because the concentration was too close to the limit of detection. The FPIA seems to rather underestimate the DCF concentration, while the ELISA tends to overestimation (on average by 32%) which is a well-known characteristic of this method. It must be said that the LC-MS/MS method determines only DCF (see the “Reference analysis by LC-MS/MS” section); the overestimation by the highly sensitive ELISA could be caused by the presence of (even less cross-reactive) DCF metabolites which occur in treated and untreated wastewater [2]. Overestimation by ELISA due to the presence of hydroxylated and conjugated metabolites, for which we cannot provide cross-reactivity data, has been described before [5]. In contrast, the underestimation (on average by 20%) by the less sensitive FPIA can be caused by the effect that it cannot detect these metabolites. Concluding, the obtained values might be more representative for the concentration of the parent compound.

Comparing the real-world data for DCF concentrations in wastewater samples of Germany, one can clearly see that diclofenac is not completely removed from the wastewater stream during the passage through the elimination stages of the wastewater treatment plants, resulting in microgram per liter concentrations that are released continuously into the receiving surface waters.

## Conclusions

Structurally different tracers to establish a fluorescence polarization immunoassay (FPIA) for diclofenac (DCF) were synthesized and tested for the first time, and a rapid, high-throughput method was developed for the detection of this emerging pollutant in wastewater. The FPIA based on the homologous tracer DCF-Ahx-AMF, including a C6 spacer, displayed satisfactory precision and sensitivity with an IC_50_ of 28 μg L^−1^ and an LOD of 2.0 μg L^−1^. The cross-reactivity of some structural analogues was found to be 15% or lower. The FPIA exhibited concordant results with LC-MS/MS in the analysis of real wastewater samples, in- and effluent. The FPIA method showed significant advantages in assay time, a sample measurement can be performed within 20 to 30 min. The satisfactory accuracy proved that the newly developed method is suitable to work as a rapid and inexpensive method for the detection of diclofenac in wastewater. Our studies on real wastewater samples showed that effluent wastewaters may still contain diclofenac concentrations which are released into surface waters. We assign the FPIA an enormous potential as a rapid monitoring or screening method for the surveillance of diclofenac or other pollutants’ inputs into the aquatic environment.

## Supplementary information

ESM 1 (PDF 563 kb)
