# Supplementary material for: Fluorescence polarization immunoassay for the determination of diclofenac in wastewater
Source: Anal Bioanal Chem. 2020 Nov 25;413(4):999–1007. doi: 10.1007/s00216-020-03058-w (PMC7813709; doi:10.1007/s00216-020-03058-w)
Supplement: Supplementary file 1 — (PDF 563 kb) [file 216_2020_3058_MOESM1_ESM.pdf]

Analytical and Bioanalytical Chemistry

Electronic Supplementary Material

Fluorescence polarization immunoassay for the determination of diclofenac in wastewater

Anna Raysyan, Robin Moerer, Bianca Coesfeld, Sergei A. Eremin, Rudolf J. Schneider

Synthesis of fluorophore-labeled diclofenac derivatives (tracers)

Three homologous fluorescent tracers to build a fluorescence polarization immunoassay for diclofenac (DCF), DCF-AMF, DCF-Ahx-AMF, DCF-Ahx-EDF, and one heterologous tracer, 5-OH-DCF-AMF, were synthesized.

DCF-Ahx (diclofenac aminohexanoic acid amide) was previously synthesized, the synthesis is described in Schmidt et al. (2017) [1]. 5-Hydroxy diclofenac (5-OH-DCF) is a commercially available metabolite of DCF.

The tracers were obtained by activation of the carboxyl group contained in the compounds DCF, DCF-Ahx, and 5-OH-DCF-AMF, and coupled with the free amino group of the respective fluorescein derivatives, 4′-(aminomethyl)fluorescein (AMF) and ethylenediamine fluoresceinthiocarbamyl (EDF) via the activated ester method with NHS/DCC [2] (see **Figs. S1-S3**). EDF was synthesized from fluorescein isothiocyanate (FITC) and ethylenediamine dihydrochloride following the method proposed by Pourfarzaneh et al. (1980) [3]. All syntheses were carried out under Argon and for most of the time in the dark.

For DCF-AMF (Fig. S1), 4.79 mg (16 µmol, 1 eq) of DCF were dissolved in 200 µL abs. *N,N*-dimethylformamide (DMF). 5.85 mg (51 µmol, 3.2 eq) of *N*-hydroxysuccinimide (NHS) and 8.90 mg (41 µmol, 2.6 eq) of *N,N'*-dicyclohexylcarbodiimide (DCC) were each dissolved in 150 µL of abs DMF and added to the DCF solution. The mixture was incubated overnight at room temperature under stirring. 60 µL of the solution containing the diclofenac NHS ester were added to 1.05 mg (2.6 µmol) AMF•HCl dissolved in 10 µL of triethylamine.

Syntheses of the other tracers were performed accordingly. For the synthesis of DCF-Ahx-AMF (Fig. S2a) and DCF-Ahx-EDF (Fig. S2b), 23 mg (200 µmol) of NHS and 41 mg DCC (200 µmol) were each dissolved in 2000 µL DMF. 3 mg (6,7 µmol, 1 eq) of DCF-Ahx was dissolved in a mixture of each 200 µL (20 µmol, 3 eq) of the NHS and DCC solutions, and incubated for 3 h at room temperature under stirring. 200 µL (< 3.3 µmol) aliquots of the solution containing the DCF-Ahx NHS ester were added to a) AMF•HCl (2.6 µmol) or b) EDF•HCl (2.2 µmol), respectively, dissolved in 10 µL of triethylamine.

For the heterologous tracer, the active ester was produced from 5-hydroxy diclofenac (Fig. S3). 5.22 mg (17 µmol, 1 eq) of 5-OH-DCF was dissolved in 200 µL abs DMF. 5.85 mg (51 µmol, 3 eq) NHS and 9 mg DCC (43 µmol, 2.5 eq) were dissolved in 250 µL abs DMF each and added to the 5-OH-DCF solution. The mixture was incubated overnight under stirring. 50 µL of the solution containing the 5-OH-DCF NHS ester formed (< 1 µmol) were added to 1.18 mg AMF•HCl (3 µmol) diluted in 10 µL of triethylamine.

All products formed a yellow-orange solution which was stirred for another 4 h before being purified by thin-layer chromatography (TLC).

TLC was performed on silica sheets (2.5 x 7.5 cm; silica gel 60 with concentration zone/without fluorescence indicator, Merck). The mobile phase was $CHCl_3$:CH_3OH:CH_3COOH (4:1:0.1, *v/v*). For each tracer, the main yellow band, clearly visible under UV light (λ = 365 nm), was collected, dissolved in ethanol, stripped from silica via filtration through a Teflon membrane syringe filter with a pore size of 0.45 µm and 17 mm

in diameter, and purified again by TLC (see retardation factors R_f in Table S1). Ethanol as solvent was evaporated, the residue finally dissolved in 100 µL methanol, stored at 4 °C and later on directly used as tracer stock solution from which dilutions (Tracer Working Solutions) in borate buffer were prepared.

The success of the synthesis was confirmed by LC-MS/MS (see Fig. S4). Mass spectra were recorded at the retention time of the largest peak in the UV trace. m/z + 1 and $+Na^+$ adduct ions for each individual synthesis product could be identified. The chromatograms showed for some compounds not a high purity, but impurities rarely interfere with FPIA analysis, thus the products were used as obtained.

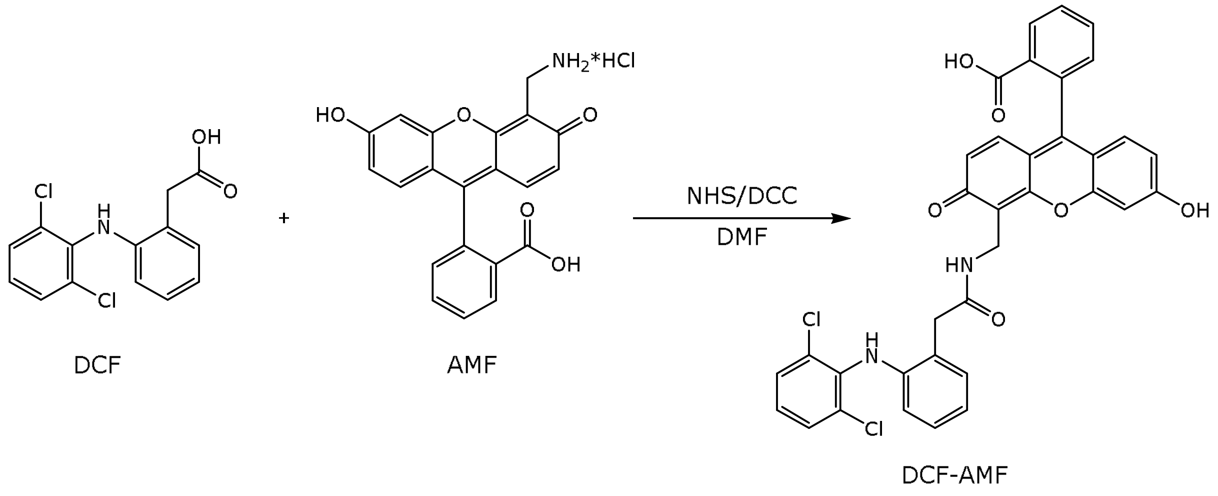

Fig. S1 Reaction scheme for the synthesis of DCF-AMF

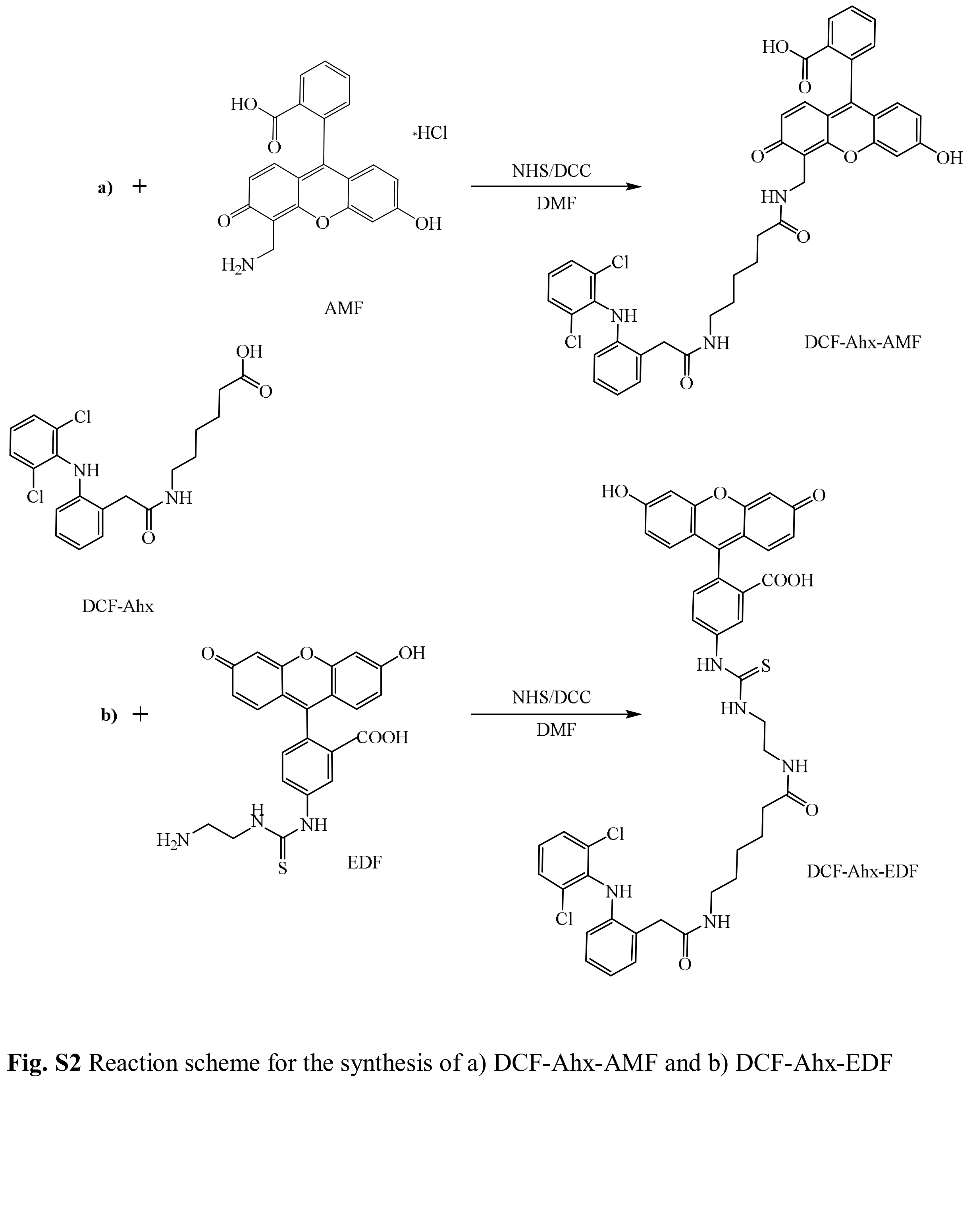

Fig. S2 Reaction scheme for the synthesis of a) DCF-Ahx-AMF and b) DCF-Ahx-EDF

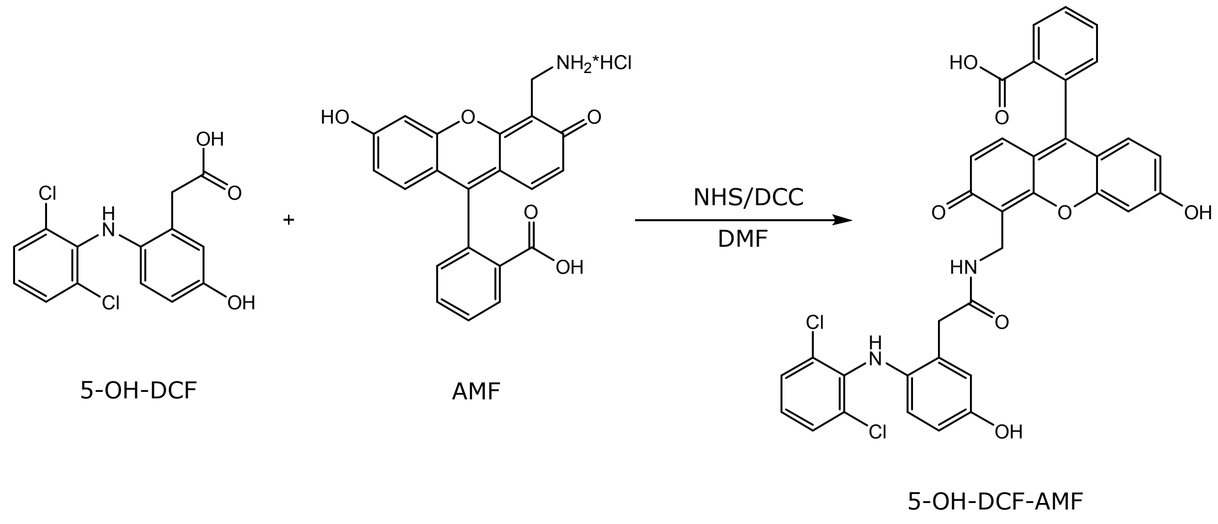

Fig. S3 Reaction scheme for the synthesis of 5-OH-DCF-AMF

Table S1 R_f values of the collected bands of the tracers during purification by TLC

Tracer	DCF-AMF	DCF-Ahx-AMF	DCF-Ahx-EDF	5-OH-DCF-AMF
TLC retardation factor R_f	0.8	0.8	0.9	0.9

Following pages:

Fig. S4 A)-E) LC-MS/MS characterization of the products of the four tracer syntheses

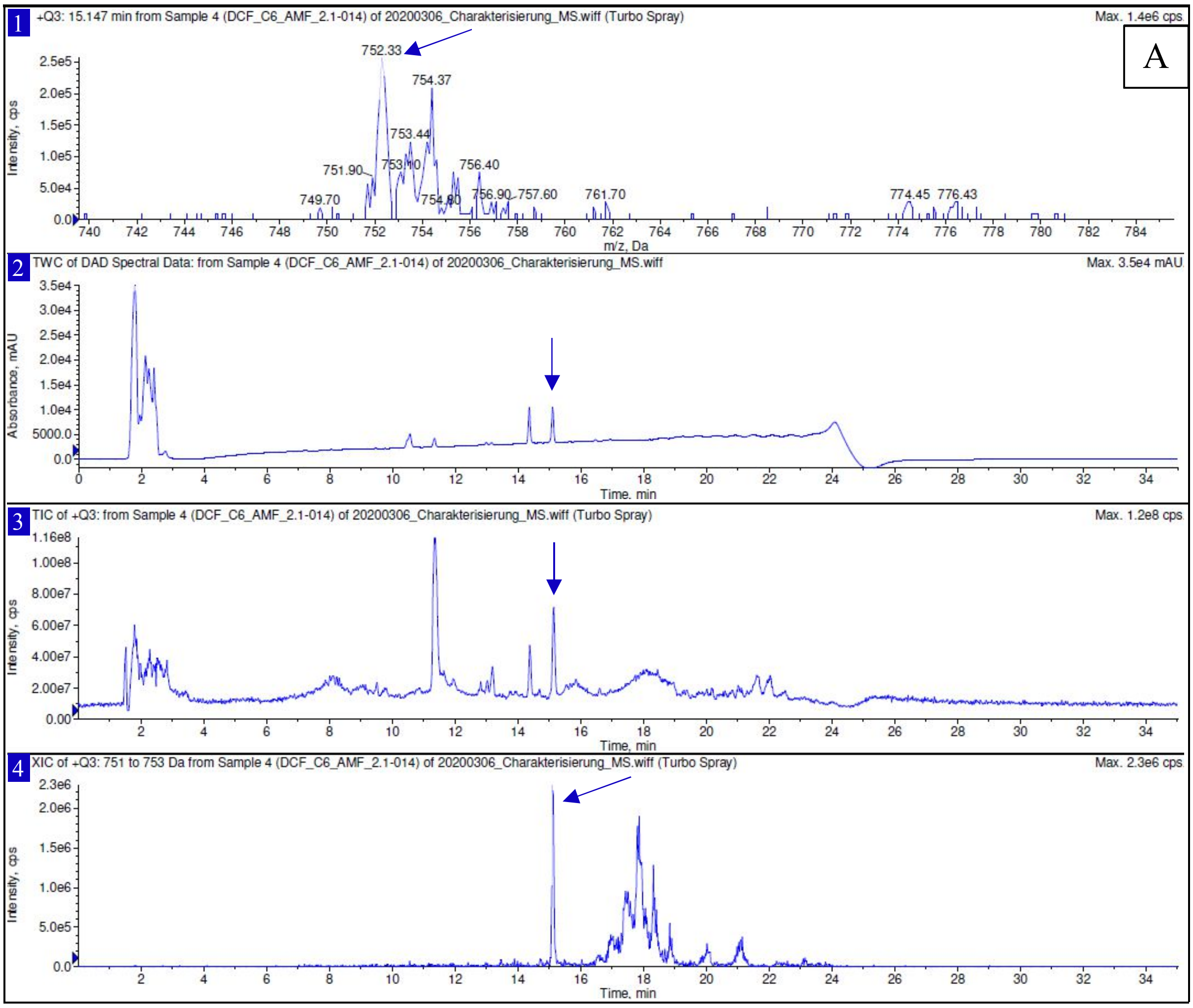

A) Chromatograms and mass spectra of products of **DCF-Ahx-AMF** synthesis (before TLC purification): 1) Mass spectrum of chromatographic peak at RT = 15.147 min (= RT of the largest peak in the DAD trace), with the largest signal at m/z = 752.33 Da. This reflects the mass of the desired product (751.2 g/mol) plus the mass of a proton (+1). 2) HPLC chromatogram, DAD trace, showing, apart from the peak at RT = 15.2 min another one at RT = 14.2 min. 3) TIC (total ion chromatogram) showing the same peaks as the DAD plus a major peak at RT = 11.2 min. 4) Extracted Ion Chromatogram (XIC, m/z = 751...753) showing the peak of the desired product at 15.1 min plus impurities in the time range 16.5 … 21.5 min.

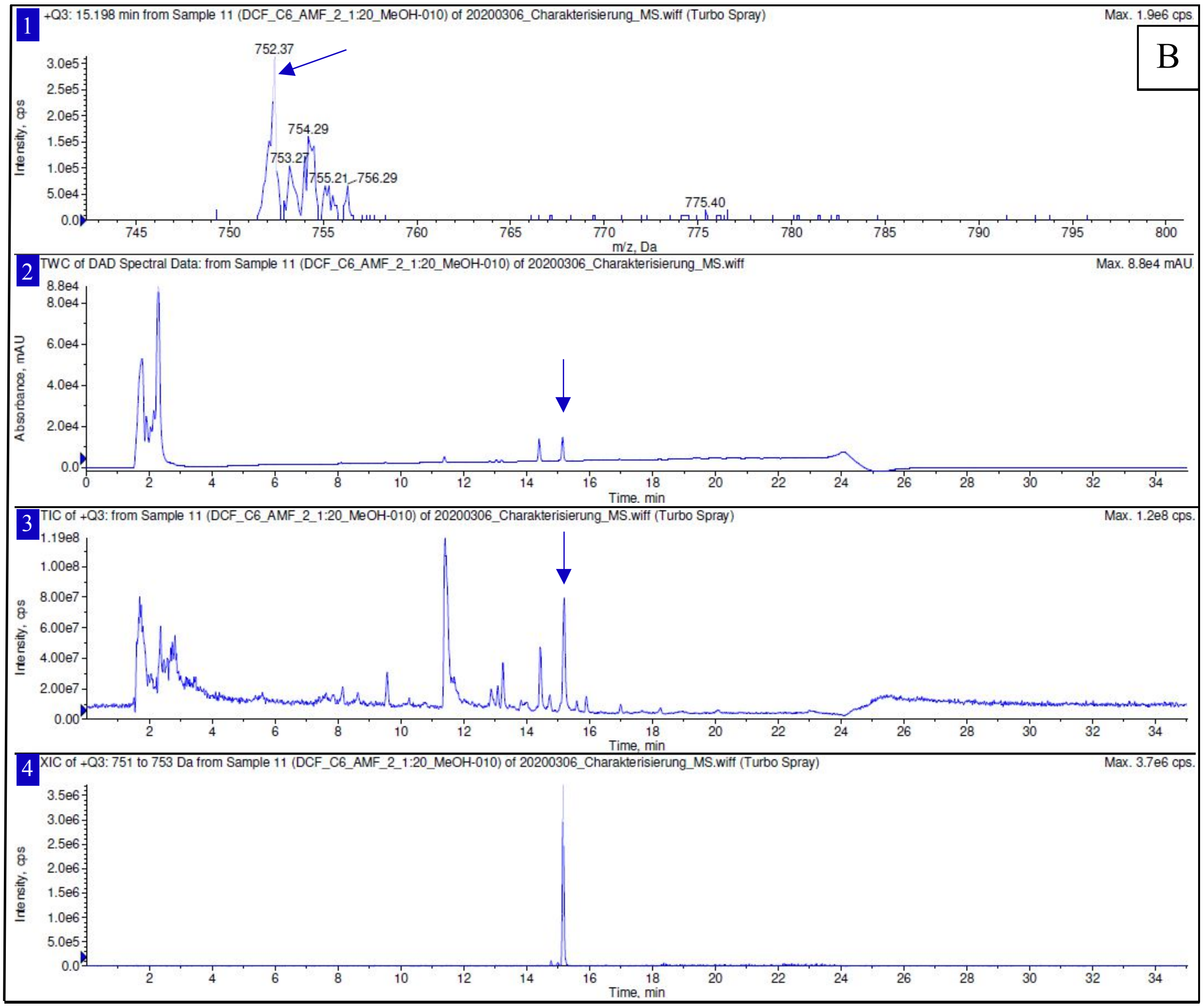

B) Chromatograms and mass spectra of products of **DCF-Ahx-AMF** synthesis (<u>after TLC purification</u>). 1) Mass spectrum of chromatographic peak at RT = 15.198 min (= RT of the largest peak in the DAD trace), with the largest signal at m/z = 752.37 Da. This reflects the mass of the desired product (751.2 g/mol) plus the mass of a proton (+1). A small signal at m/z = 775.40 corresponds to the mass of the product plus one sodium ion (+22.9). 2) HPLC chromatogram, DAD trace, showing two peaks, the peak at 15.2 min being the product. 3) TIC chromatogram showing the peak of the desired product at RT = 15.2 min. 4) XIC (ion range 751 … 753 Da) showing the mass trace of DCF-Ahx-AMF at the correct retention time.

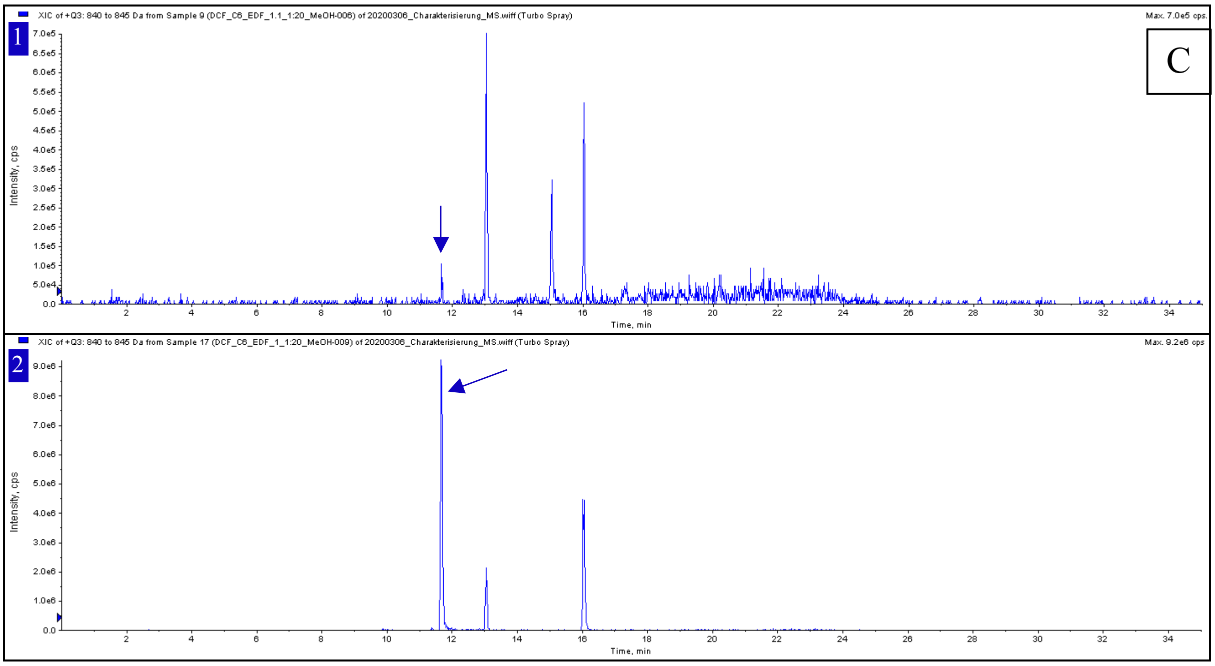

C) Chromatograms of products of **DCF-Ahx-EDF** (1) before and (2) after TLC purification: 1) The XIC chromatogram, constructed from extracted ions in the m/z range of 840 … 845 (mass of desired product is 839.2 g/mol) shows, besides the peak at RT = 11.9 min, three major impurities that are detected around RT 13, 15 and 16 min. From the relative peak areas it can be assumed that DCF-Ahx-EDF is only a minor component among the products formed. 2) The XIC of the chromatographic run after purification reveals that the desired product is now the main component in the mixture.

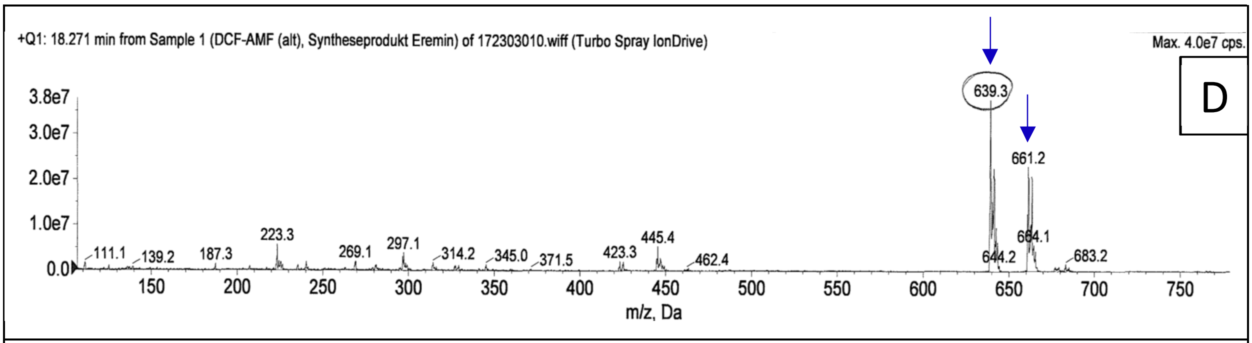

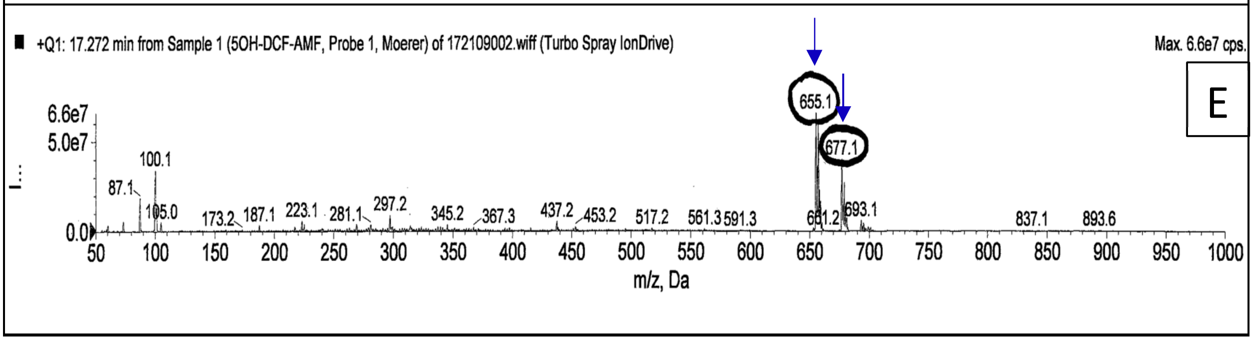

D) Synthesis of DCF-AMF: Mass spectrum of chromatographic peak at RT = 18.271 min (= RT of the largest peak in the UV trace), with m/z = 639.3 Da und 661.2 Da. This represents the mass of the desired product (638.3 g/mol) plus the mass of one proton (+1) resp. one sodium ion (+22.9). The spectrum shows some minor ions originating from side products.

E) Synthesis of 5-OH-DCF-AMF: Mass spectrum of chromatographic peak at RT = 17.272 min (= RT of the largest peak in the UV trace), with two low-intensity signals at m/z = 655.1 Da and 677.1 Da. These reflect the masses of the desired product (654.1 g/mol) plus the mass of one proton (+1) resp. one sodium ion (+22.9). The analysis shows that there are no major impurities in the reaction mixture.

Additional data on real wastewater samples

Table S2 Total organic carbon content (TOC) and pH of the wastewater samples listed in Table S2

Sample No.	TOC (mg L^{-1})	pH
1	105	7.6
2	101	7.5
3	111	7.2
4	20.0	7.7
5	26.1	7.1
6	30.2	6.8

References

1. Schmidt S, Hanelt S, Canitz C, Hoffmann H, Garbe LA, Schneider RJ (2017) Synthetic Strategies for the Modification of Diclofenac. Synlett 28 (15):1984-1989. https://doi:10.1055/s-0036-1588858

2. Bondarenko AP, Eremin SA (2012) Determination of zearalenone and ochratoxin A mycotoxins in grain by fluorescence polarization immunoassay. J Anal Chem 67 (9):790-794. https://doi:10.1134/S1061934812090031

3. Pourfarzaneh M, White GW, Landon J, Smith DS (1980) Cortisol directly determined in serum by fluoroimmunoassay with magnetizable solid phase. Clin Chem 26 (6):730-733
